# Immunological aspects of central neurodegeneration

**DOI:** 10.1038/s41421-024-00666-z

**Published:** 2024-04-09

**Authors:** Mireia Niso-Santano, José M. Fuentes, Lorenzo Galluzzi

**Affiliations:** 1 Departamento de Bioquímica y Biología Molecular y Genética, Facultad de Enfermería y Terapia Ocupacional, Universidad de Extremadura, Cáceres, Spain; 2 Centro de Investigación Biomédica en Red en Enfermedades Neurodegenerativas-Instituto de Salud Carlos III (CIBER-CIBERNED-ISCIII), Madrid, Spain; 3 Instituto Universitario de Investigación Biosanitaria de Extremadura (INUBE), Cáceres, Spain; 4 Department of Radiation Oncology, Weill Cornell Medical College, New York, NY USA; 5 Sandra and Edward Meyer Cancer Center, New York, NY USA; 6 Caryl and Israel Englander Institute for Precision Medicine, New York, NY USA

**Keywords:** Mechanisms of disease, Innate immunity

## Abstract

The etiology of various neurodegenerative disorders that mainly affect the central nervous system including (but not limited to) Alzheimer’s disease, Parkinson’s disease and Huntington’s disease has classically been attributed to neuronal defects that culminate with the loss of specific neuronal populations. However, accumulating evidence suggests that numerous immune effector cells and the products thereof (including cytokines and other soluble mediators) have a major impact on the pathogenesis and/or severity of these and other neurodegenerative syndromes. These observations not only add to our understanding of neurodegenerative conditions but also imply that (at least in some cases) therapeutic strategies targeting immune cells or their products may mediate clinically relevant neuroprotective effects. Here, we critically discuss immunological mechanisms of central neurodegeneration and propose potential strategies to correct neurodegeneration-associated immunological dysfunction with therapeutic purposes.

## Introduction

Neurodegenerative disorders (NDs) are a heterogeneous group of pathologies that is characterized by the progressive degeneration of the structure and function of the central or peripheral nervous system^1–3^. Severity and propensity to progression not only vary across different NDs, but also across patients presenting with the same pathology, which considerably complicates the effectiveness of therapeutic interventions^4^. In fact, no disease-modifying therapeutic strategies are currently available for most NDs^5–7^, perhaps with the exception of lecanemab, a controversial agent recently licensed by the US Food and Drug Administration (FDA) for the therapy of early Alzheimer’s disease (AD)^8^.

Common NDs affecting the central nervous system (CNS) including but not limited to AD, Parkinson’s disease (PD), and Huntington’s disease (HD) are generally attributed to intracellular defects in specific neuronal populations that over time compromise cellular homeostasis (often along with the progressive accumulation of specific proteins within neurons or in their microenvironment), ultimately culminating with widespread or localized neuronal loss coupled with clinical cognitive, behavioral or motor symptoms^1,3,9^. In line with this notion, while juvenile variants of central NDs exist^10^, the prevalence and severity of most NDs increases with age^11^. Moreover, a number of genetic mutations affecting intracellular processes have been associated with an increased risk for central NDs^12,13^. For instance, mutations in amyloid beta precursor protein (*APP*), which encodes the precursor of the AD pathognomonic amyloid beta (Aβ) peptide, is associated with an increased risk for AD^1^. Similarly, most cases of HD are associated with mutations in huntingtin (*HTT*), which results in the generation of an altered HTT variant with pathogenic features^9^. That said, some NDs are not necessarily associated with genetic predisposition^14,15^, suggesting that additional factors may be involved in their pathogenesis.

A number of non-genetic factors have been proposed as contributors to the pathogenesis of NDs, including (but not limited to): (1) the abundance and composition of the gut microbiome^16,17^, (2) a history of infection with viral or bacterial pathogens^18^, (3) the existence of subjacent or overt cardiovascular and metabolic disorders^19^, as well as (4) dysfunctions of the innate or adaptive immune system^20,21^ (Box 1). Recent data from other biomedical disciplines point indeed to an underappreciated contribution of dysregulated immunity to the pathogenesis of disorders that have classically been attributed to cell-intrinsic mechanisms, including various cardiovascular conditions^22,23^ and cancer^24^. Moreover, microglial cells, which play a major role in the development of multiple NDs (see below), are brain-resident cells of hematopoietic origin with macrophage-like functions, which render them preferential interactors of other potentially pathogenic immune cells^25^.

Here, we discuss immunological mechanisms that promote central neurodegeneration in the context of human NDs and propose potential strategies to correct neurodegeneration-associated immunological dysfunctions with therapeutic purposes. Conversely, immunological aspects of peripheric NDs including multiple sclerosis and amyotrophic lateral sclerosis have been extensively covered elsewhere^26,27^, and hence will not be covered in this review.

Box 1 Principles of innate and adaptive immunity The mammalian immune system is generally organized around two mutually interactive arms commonly referred to as innate and adaptive immunity^183,184^. These two systems mainly differ from each other in that: (1) while innate immunity generally operates as a first, broad mechanism of defence against perturbations of cellular or organismal homeostasis, adaptive immunity is elicited at a subsequent step and involves the specific recognition of antigenic determinants^185^; (2) while innate immune cells comprise both myeloid (e.g., macrophages, neutrophils, dendritic cells) and lymphoid (e.g., NK cells), adaptive immunity is only mediated by lymphocytes, namely T cells and B cells^186^; (3) while innate immune responses are only associated with some degree of training (meaning that some innate immune cells can acquire improved reactivity upon exposure to an activating stimulus)^187^, adaptive immune responses most often result in the formation of robust antigen-specific immunological memory (implying that T cells and B cells recognizing their cognate antigen generate a memory cell pool that can persist in the organism for decades)^188^. Such a memory, which involves a soluble, antibody-dependent, as well as a cellular, T cell-mediated, component, underlies the ability of adaptive immune cells to mount rapid and strong responses to subsequent challenges with the same antigenic determinants, forming the basis for prophylactic vaccination^189^. Notably, innate immunity has evolved earlier than adaptive immunity, and from a molecular perspective relies on activatory or inhibitory receptors that reside in various subcellular compartments (including but not limited to the plasma membrane, cytosol and endosomes) and recognize exogenous or endogenous molecules^190^. As an example, the activation of innate lymphoid cells including NK cells is mainly regulated by positive and negative inputs from surface-exposed receptors of the natural cytotoxicity receptor (NCR) and killer Ig-like receptor (KIR) family, respectively^191,192^. Conversely, the activation of B and T lymphocytes is generally initiated by a plasma-membrane associated receptor that is generated by the recombination of a genetic locus resulting in extraordinary diversity, de facto endowing the adaptive immune system with specificity against virtually any molecular structure (including mammalian molecules)^193^. Thus, modern adaptive immunity in mammals has co-evolved with a number of mechanisms that prevent widespread autoimmunity, including (but not limited to): (1) a safeguard system that impedes B and T cell activation in the absence of positive signals other beyond B-cell receptor (BCR) and T-cell receptor (TCR) engagement; and (2) the elimination or inhibition of autoreactive B cell and T cell clones as mediated by both central (thymic) and peripheral tolerance^194^. Importantly, both innate and adaptive immune mechanisms have been shown to contribute to neurodegeneration.

## Alzheimer’s disease

AD, which is the most common cause of dementia amongst the elderly, is characterized by a progressive loss of cognitive functions and memory associated with the accumulation of extracellular Aβ plaques and intracellular microtubule associated protein tau (MAPT, best known as tau) neurofibrillary tangles^1^.

AD has been consistently linked to genetic alterations in apolipoprotein E (*APOE*), encoding a protein involved in lipid metabolism^28^, as well as in a number of genes involved in innate immunity and microglia activation, notably triggering receptor expressed on myeloid cells 2 (*TREM2*), encoding a plasma membrane receptor that promotes phagocytosis^29–31^; complement C3b/C4b receptor 1 (*CR1*), encoding a complement component^32^; *CD33*^33,34^; major histocompatibility complex, class II, DR beta 1 (*HLA-DRB1*), *HLA-DRB5* and *HLA-DR15*, all encoding MHC molecules^35,36^; protein tyrosine kinase 2 beta (*PTK2B*), inositol polyphosphate-5-phosphatase D (*INPP5D*), and phospholipase C gamma 2 (*PLCG2*), all encoding intracellular signal transducers^31,35^; and ABI family member 3 (*ABI3*), encoding an adaptor protein^31^, pointing to microglia-driven neuroinflammation as a major pathogenic determinant of this ND.

Abundant preclinical data suggest that microglial activation may have a context- and disease stage-dependent effect on the progression of AD. On the one hand, inhibition of TREM2 by genetic or pharmacological strategies has been associated with limited microglial activity and neuroprotection in various mouse models of AD and tau pathology, including mice expressing five protein variants associated with familial AD (i.e., 5XFAD mice)^37–39^, mice expressing a human pathogenic variant of tau (so-called P301S mice)^40,41^, P301S mice expressing human AD-linked variant of APOE (i.e., APOE4)^42^, as well as 5XFAD mice intracerebrally administered with sarkosyl-insoluble tau aggregates isolated from the frontal cortex of human AD brain tissue^43–45^. However, TREM2 overexpression as well as increased TREM2 activation upon inhibition of membrane shedding have also been linked with improved biochemical and cognitive manifestations of AD in rodent models of the disease^46,47^. Thus, TREM2 appears to influence AD progression in a complex manner that may involve an initial beneficial impact related to the degradation of amyloid plaques and a subsequent detrimental impact linked to neuroinflammation.

To add yet another layer of complexity, in at least some AD models, while *Trem2* haploinsufficiency appears to aggravate tau pathology in mice, complete *Trem2* loss reportedly limits tau-driven microglial activation and atrophy^48^. Whether these apparently contradictory findings may relate to the differential activation of inflammatory responses in cells other than the microglia, such as oligodendrocytes^49^, remains to be further investigated. The existence of different microglial clusters as documented by modern single-cell sequencing technologies may also explain, at least in part, the apparently context-dependent impact of TREM2 on AD progression in mice. For instance, a novel type of neurodegenerative disease-associated microglia (DAM) has been shown to mediate neuroprotective effects in 5XFAD mice via a mechanism that at least initially involves TREM2 signalling^50^. Similar results have been obtained in mice expressing pathogenic APP variants (namely, App^NL-G-F^ mice), a scenario in which APOE expression by a DAM-like microglia was positively associated with an improved clearance of Aβ plaques^51^. In line with this notion, APOE has been shown to promote AD progression in mice bearing pathogenic APP and presenilin 1 (PSEN1, best known as PS1) variants (namely, APP-PS1 mice)^52^ and P301S mice^53^, a neurodegenerative mechanism mapping to the subset of microglia that exhibit a common disease-associated phenotype in mice and humans^52,54^.

Importantly, at least part of these APOE-associated mechanisms leading to neurodegeneration have been mapped to the inability of AD-linked APOE variants to preserve homeostatic (tolerogenic) microglial functions, culminating with the expression of multiple pro-inflammatory transcription factors^52^. In line with this possibility, APOE expression in astrocytes favors the polarization of microglia towards a DAM state in P301S mice^54^. Moreover, neuronal expression of APOE4 drives a specific DAM subset with potent neurodegenerative effects in an APOE4-expressing tauopathy mouse model, an effect that can be circumvented via neuron-specific *ApoE* deletion^55^. That said, human AD-associated microglia (HAM) as characterized by RNA sequencing from frozen samples of frontal cortex from AD-affected individuals appears to exhibit little transcriptional resemblance with the DAM as identified in mouse AD models^56^, with the notable exception of a common APOE overexpression^57^. Moreover, the HAM signature appears to be detectable also in patients with non-AD NDs, which is not the case for the DAM transcriptional profile^56^.

Thus, while initial microglial engagement has beneficial effects on AD progression at least partly emerging from the clearance of Aβ deposits, the overactivation of microglia has consistently been attributed a neurotoxic activity related to accrued oxidative stress and inflammation. Interestingly, such overactivation has been reported to culminate with microglial dystrophy^58–60^, a phenotype that appears to be common to multiple NDs beyond AD^61–63^. Additional work is required to identify strategies to finely modulate microglial functions for the treatment of AD.

Importantly, pro-inflammatory molecules secreted by the activated microglia including interleukin 1A (IL1A), tumor necrosis factor (TNF) and complement C1q A chain (C1QA) have been shown to promote astrocyte activation, resulting not only in a loss of phagocytic and synapse-promoting activity, but also in the secretion of neurotoxins that promotes neuronal and oligodendrocyte death^64^. In line with a pathogenic role for astrocytes in AD, astrocytic tau accumulation in the dentate gyrus has been shown to promote neuronal dysfunction and memory deficits in mice^65^. Moreover, reactive astrocytes have been identified in early stages of human AD and appear to be present ubiquitously throughout disease progression^66^. Moreover, while mildly reactive astrocytes have limited neurotoxic potential, severe astrocyte activation has been linked to neurotoxic and ultimately pathogenic hydrogen peroxide production via monoamine oxidase B (MAOB), at least in mice^67^. That said, astrocytes resemble microglia in being highly heterogenous and exhibiting an age-dependent decline in neuroprotective activity^68,69^. In line with this notion, a disease-associated astrocyte (DAA) transcriptional profile as identified in multiple rodent models of AD appears to emerge early during disease progression and exacerbate over time, a pathogenic progression also observed in aged wild-type mice and humans^70^.

While transcriptionally different from their murine counterparts^64^, DAAs from individuals with AD have been subclassified into 8 different clusters, one of which exhibits signatures of immune signaling including transforming growth factor beta 1 (TGFB1) activation^57^. Of note, human DAAs overexpress APOE as well as glypican 4 (GPC4), a secreted factor that has been detected in post-mortem brains from patients with APOE4-associated AD^70,71^ and may contribute to disease progression upon direct interaction with APOE4 and consequent tau hyperphosphorylation^57^. Supporting the pathogenic role of this mechanism, *Apoe4* deletion from astrocytes limits disease progression in P301S mice^54^. Thus, pro-inflammatory signaling elicited by microglia may promote AD progression also via astrocytes.

Yet another non-neuronal cell type involved in AD are so-called disease-associated oligodendrocytes (DOLs). A study integrating multiple datasets from mouse models of NDs and post-mortem data from ND patients identified three different oligodendrocyte activation states: disease-associated 1 (DA1), DA2 and interferon (IFN)-associated^72^. Intriguingly, not only IFN-associated DOLs but also DA1 DOLs exhibit upregulation of multiple genes involved in innate and adaptive immunity, including multiple cytokine- and complement-encoding genes as well as genes coding for MHC Class I and Class II molecules^72^. At least some of these DOL signatures are not restricted to AD, but can also be documented in other NDs and neuroinflammatory disorders, suggesting a common response to severe pathological conditions^73^. Of note, DOLs have TREM2-independent transcriptional responses to neurodegenerative conditions that resembles those of DAAs^49^, including the overexpression of stress-responsive proteins such as serpin family A member 3 (SERPINA3)^73^. That said the transcriptional profile of DOLs from individuals with AD considerably differs from that of mouse AD models^72^.

Importantly, not only brain-resident cells engaging in innate immune signaling, but also newly recruited immune cells have been shown to contribute to AD pathogenesis. For instance, neutrophil infiltration has been documented in individuals with various NDs including AD, a finding that has been mechanistically linked with reduced cerebral blood flow in APP-PS1 mice, 5XFAD mice, as well as mice expressing pathogenic variants of APP, PS1 and tau (namely, 3xTg mice). In these models, blood flow and short-term memory function rapidly improve when cerebral perfusion is restored by preventing neutrophil adhesion^74,75^. Along similar lines, several studies have demonstrated infiltration of the brain parenchyma by CD4^+^ and CD8^+^ T cells (which orchestrate and execute antigen-specific immune responses, respectively) in patients with AD^76,77^ and in animal models of the disease^49,78^. In this setting, extravascular cytotoxic CD8^+^ T cell abundance appears to correlate with disease stage, indicating a potential role for adaptive immunity in the pathogenesis of AD^79–81^. Similar findings have been obtained in APP-PS1 and 5XFAD mice^81^. Importantly, in this latter setting CD8^+^ T cell infiltration of the brain parenchyma could be mechanistically linked to the microglia, and not only CD8^+^ T cell depletion but also interference with CD8^+^ functions by interferon gamma (IFNG) or programmed cell death 1 (PDCD1, best known as PD-1) blockage mediated considerable neuroprotective effects^81^, potentially linked to the reversal of CD8^+^ T cell exhaustion^82^. Further supporting a link between CD8^+^ T cell activity and the pathogenesis of AD, peripheral blood mononuclear cells (PBMCs) from patients with AD are enriched in CD8^+^CD45RA^+^ T effector memory (T_EMRA_) cells displaying transcriptional signatures of activation, and their abundance negatively correlated with cognition^83^.

Conversely, the actual impact of helper CD4^+^ T cells on AD progression remains to be formally established. Indeed, administration of Aβ-specific type 1 (T_H_1) and type 17 (T_H_17) CD4^+^ T cells (two specialized populations of CD4^+^ T cells characterized by specific secretory profiles) reportedly exacerbates memory impairment and amyloid deposition in APP-PS1 mice^84^. Moreover, CD4^+^ T cell depletion appears to improve Aβ clearance and cognitive performance in 5XFAD mice^85^. However, CD4^+^ T cell depletion has also been associated with accelerated cognitive decline with no impact on amyloid pathology in APP-PS1 mice^86^. At least theoretically, these apparently contrasting findings may relate to the considerable phenotypic and functional diversity of CD4^+^ T cells, encompassing a highly reactive compartment as well as immunosuppressive subpopulations such as CD4^+^CD25^+^FOXP3^+^ regulatory T (T_REG_) cells^87,88^. Indeed, T_REG_ cells have consistently been shown to limit AD progression in mouse models of the disease^89–91^.

Interestingly, multiple microglia–T cell interactions have been documented in patients with AD and/or mouse models thereof, including (but not limited to) TCR responses to MHC Class II-restricted peptides^92^. That said, single-cell analyses of T cells infiltrating the brain of mice affected by amyloid, tau or combined (amyloid and tau) pathology suggest that T cell reactivity is not influenced by TREM2 expression in the DAM^49^. Taken together, these observations suggest that while the DAM may drive pathogenic T cell responses during AD progression, such responses (at least initially) may not impinge on the APOE-TREM2 signalling axis. Recently, B cells (a population of lymphoid cells specialized in antigen presentation and antibody production) have also been mechanistically implicated in the pathogenesis of AD^93^. Specifically, 3×Tg mice have been shown to exhibit not only an expanded B cell comportment in the periphery, but also accrued B cell accumulation in the brain parenchyma associated with immunoglobulin deposition at amyloid plaques^93^. In the same setting, B cell depletion at disease onset appears to reduce amyloid accumulation, limit hippocampal microglial activation and overall decelerate disease progression^93^.

**Fig. 1:**
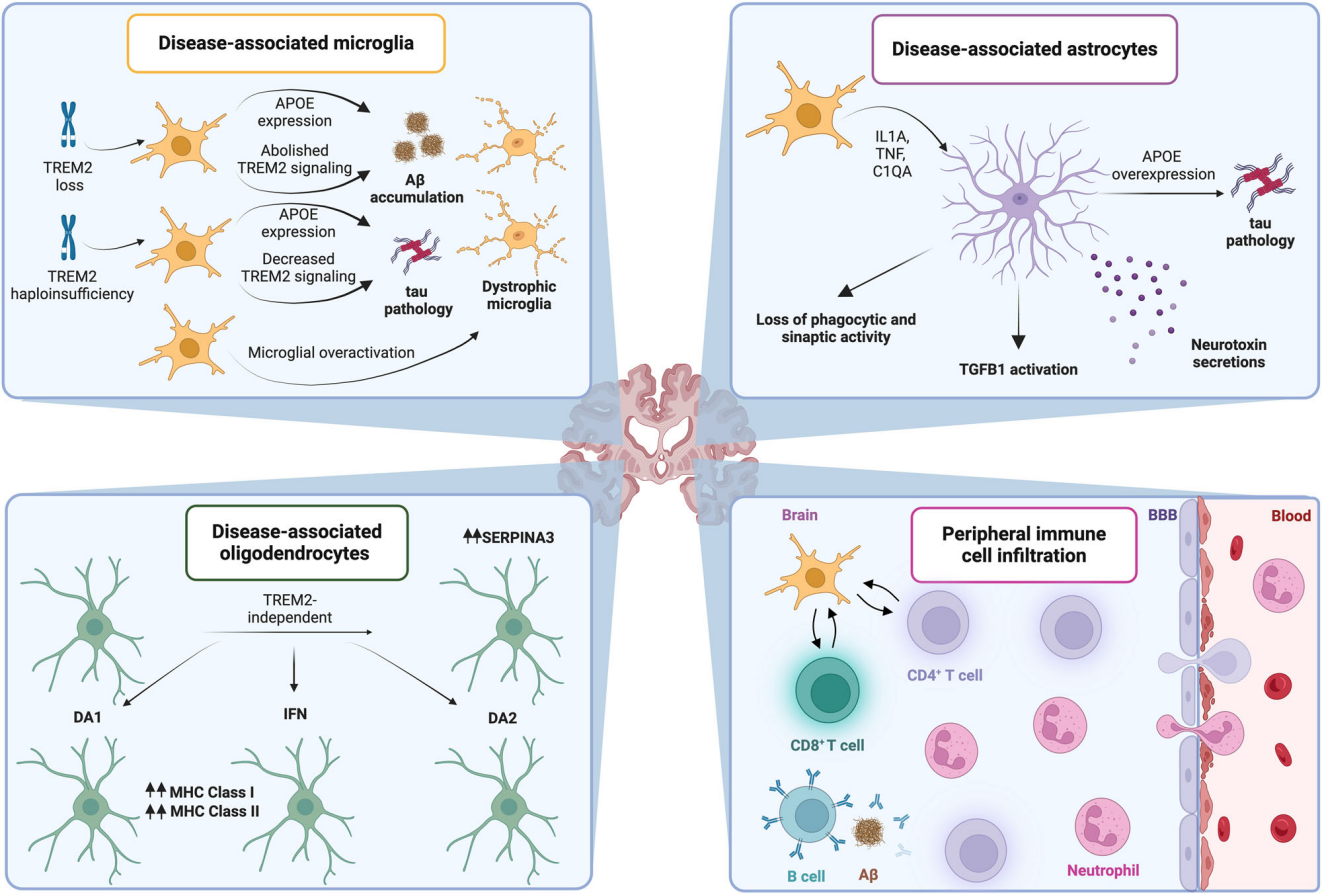
Immunological aspects of Alzheimer’s disease. Alzheimer’s disease (AD) develops in the context of complex immunological alterations that involve not only microglial cells, with a major role for altered apolipoprotein E (APOE) and triggering receptor expressed on myeloid cells 2 (TREM2) signalling, but also astrocytes and oligodendrocytes, culminating with a neuroinflammatory state associated with immune cell infiltration from the periphery. Aβ amyloid beta, BBB blood-brain barrier, C1QA complement C1q A chain, DA disease-associated, IFN interferon, IL1A interleukin 1A, SERPINA3 serpin family A member 3, tau (microtubule-associated protein tau, MAPT), TGFB1 transforming growth factor beta 1, TNF tumor necrosis factor. Created with BioRender.com.

In summary, the pathogenesis of AD involves a complex neuroinflammatory reaction involving brain-resident cells as well as newly recruited immune cells that ultimately promote neurotoxicity coupled to cognitive disorders (Fig. 1).

## Parkinson’s disease

PD is the most prevalent ND that results in disordered movement, affecting 6–7 million individuals worldwide^3^. While most PD cases are idiopathic, a familial form of the disease has been associated with mutations in > 20 genes, including parkin RBR E3 ubiquitin protein ligase (*PRKN*), PTEN-induced kinase 1 (*PINK1*), both of which encode component of the molecular apparatus that removes dysfunctional mitochondria (so-called mitophagy)^94^, leucine-rich repeat kinase 2 (*LRRK2*), encoding a multifunctional kinase, and synuclein alpha (*SNCA*)^95^. The main pathological feature of PD is the degeneration of dopaminergic neurons in the substantia nigra (SN) involving the intraneuronal accumulation of SNCA aggregates called Lewy bodies^3^. Of note, SNCA aggregates have also been documented in the gastrointestinal tract of PD patients up to 20 years prior to their diagnosis^96^, and the administration of preformed SNCA fibrils into the duodenal and pyloric muscularis layer promotes PD development in mice^97^. These observations point to the existence of a gut-to-brain axis that contributes to the spread of pathogenic Lewy bodies to the central nervous system.

While neuronal dysfunction coupled with oxidative stress has a major role in the pathogenesis of PD, variations in numerous genes encoding key components of the innate and adaptive immune system have been associated with an increased risk for PD, including an MHC Class II haplotype that is displayed by ~15% of the population (namely, *HLA-DRB1*)^98^. Moreover, multiple genetic loci associated with an increased risk for PD appear to also predispose to some autoimmune and inflammatory diseases, such as Crohn’s disease^99^. Of note, PD patients often exhibit elevations in the circulating or cerebrospinal levels of cytokines such as TNF, interleukin 1 beta (IL1B), IL2 and IL10^100,101^. In line with this notion, microglial activation is a characteristic finding in the SN of post-mortem brains from patients with PD feature of the substantia nigra in post-mortem human brains with PD^77,102^, and the active microglia has been shown to actively engage in pro-inflammatory signalling via various molecular platform including (but not limited to) the NF-κB, inflammasome, JAK/STAT and Toll-like receptor (TLR) signaling^103–105^, at least in some settings as a direct consequence of tau accumulation^106^. Importantly, multiple pharmacological strategies aimed at interrupting these signal transduction cascades have been shown to decelerate PD progression in animal models, including rats intracerebrally administered with an SNCA-encoding adenovirus or treated with the PD-inducer rotenone^103–105^. Together with the fact that significant microgliosis has been documented in areas not showing significant neuronal death in post-mortem brains from patients with PD^107–109^ and with kinetic data from rodent models of PD^110^, these findings suggest that inflammatory microglial activation precedes and promotes the demise of dopaminergic neurons that characterize PD. At least in part, such a neurotoxic response involves the microglia-driven conversion of astrocytes to a pathogenic state, as mechanistically demonstrated with a small molecule that prevents this conversion (i.e., NLY01) in mice expressing a pathogenic variant of SNCA or administered intracerebrally with preformed SNCA fibrils^111^.

Further supporting a link between inflammation and the pathogenesis of PD, SNCA has been shown to promote microglial activation in an MHC Class II-dependent manner, culminating with the initiation of a pathogenic CD4^+^ T cell response^112^. In line with this notion, SNCA overexpression in the mouse midbrain results in the upregulation of MHC Class II molecules on myeloid cells coupled with abundant infiltration of IFNG-producing CD4^+^ and CD8^+^ T cells^113^, a process that at least in part involves so-called border associated macrophages (BAMs)^114^. However, while the absence of CD3^+^ T cells or CD4^+^ T cells reportedly decelerates PD progression and ameliorate behavioral symptoms in mice treated with the PD driver 1-methyl-4-phenyl-1,2,3,6-tetrahydropyridine (MPTP), the same does not hold true for the selective absence of CD8^+^ T cells^102^. That said, CD8^+^ T cell infiltration has been documented in the SN from pre-symptomatic PD patients, de facto preceding dopaminergic neuron loss and SNCA pathology^115^. Moreover, patients with overt PD exhibit: (1) a reduction in the levels of circulating naïve T cells and T_REG_ cells^116^, (2) an increase in the ratio of IFNG- over IL4-producing CD4^+^ T cells in the periphery^117^, and (3) circulating T cells responding to SNCA-derived peptides^118^, an autoreactivity that appears to develop even prior to clinical manifestations of the disease^119^. Finally, results from *Snca*^*−/−*^ mice indicate that SNCA is also required for the development of normal inflammatory and antigen-specific responses to intraperitoneal bacteria^120^, further strengthening the links between PD and immunity.

**Fig. 2:**
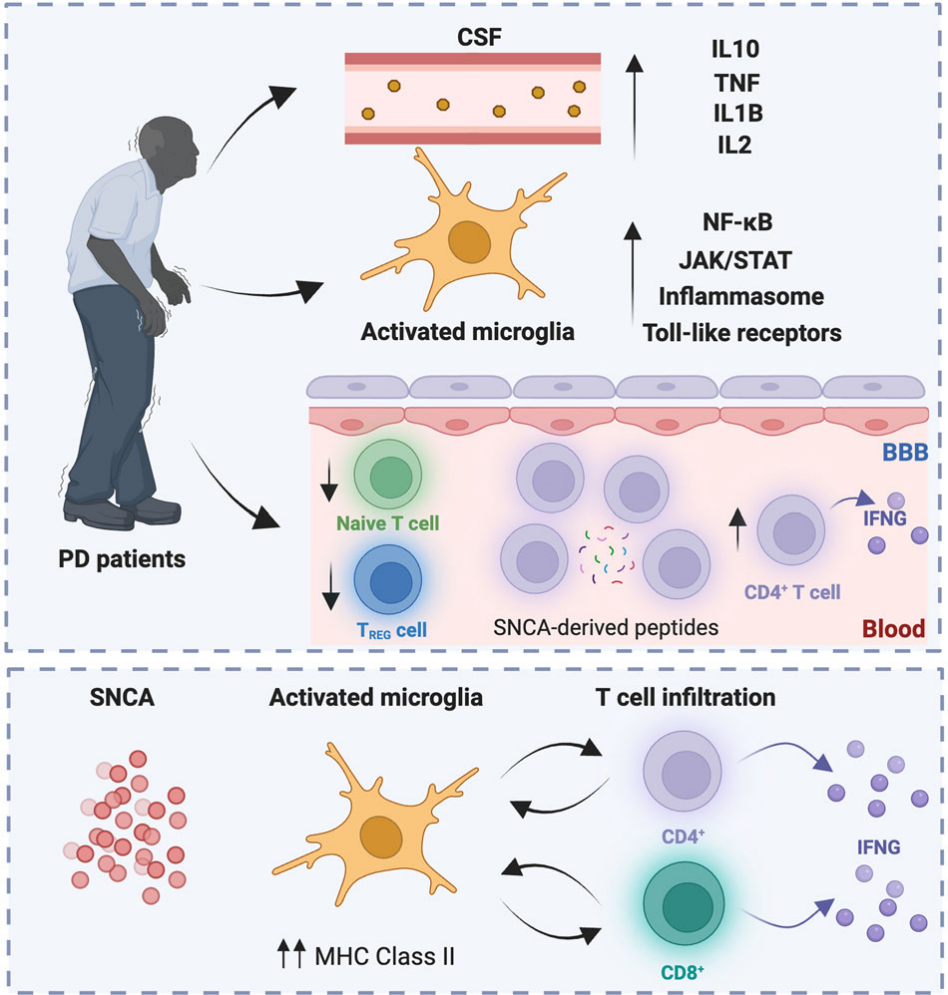
Immunological aspects of Parkinson’s disease. Patients with Parkinson’s disease (PD) exhibit synuclein alpha (SNCA)-related microglial activation coupled with the initiation of multiple signalling pathways that result in the abundant secretion of pro-inflammatory cytokines in the cerebrospinal fluid (CSF). Such cytokines promote the recruitment of immune cells that contribute to neuroinflammation by secreting proinflammatory mediators such as interferon gamma (IFNG). BBB blood brain barrier, IL interleukin, TNF tumor necrosis factor, T_REG_ regulatory T. Created with BioRender.com.

In summary, PD appears to involve a variety of innate and adaptive immune processes that culminate with the loss of dopaminergic neurons in the SN and the consequent motor symptoms (Fig. 2).

## Huntington’s disease

HD is an autosomal dominant neurological disorder caused by an aberrant expansion of CAG triplets in exon 1 of *HTT*, resulting in a long polyglutamine tract that disrupts HTT cellular functions and causes widespread neurotoxicity starting from the neostriatum^3^. Clinical HD manifestations include progressive cognitive, motor and behavioral impairments^3^.

Similar to AD and PD, HD is also characterized by microgliosis, both in humans^121–123^ and in mouse models of the disease such as R6/2 mice^124–126^. Moreover, HD resembles AD and PD in that inflammatory microglial activation constitutes an early event in the pathogenesis of disease as it has been documented in post-mortem brain samples from patients with pre-symptomatic HD^126,127^. Of note, HD-associated microgliosis has also been linked with alterations in circulating myeloid cells and cytokines, and at least some of these changes could be detected in mutant *HTT* carriers prior to clinical manifestations of the disease^128,129^.

Several lines of evidence implicate HTT-driven inflammatory microglial activation in the pathogenesis of HD. First, microglial depletion using an inhibitor of colony-stimulating factor 1 receptor (CSF1R) reduces mutant HTT accumulation and prevents striatal atrophy in R6/2 mice^130^. Second, the establishment of mouse chimeras incorporating human microglial cells expressing mutant HTT promotes motor impairment and neuronal dysfunction in the striatum, while the contrary is true when the human microglia expresses wild-type HTT^131,132^. Third, mutant HTT expression in microglial cells is sufficient to drive microgliosis and elicit neurodegeneration^133^.

HD-associated HTT mutations also affect astrocytic and oligodendrocytic cells. For instance, mutant HTT expression in microglia has been shown to deregulate the expression of multiple cell lineage-specific genes in astrocytes from R6/2 mice and zQ175 mice (another mouse model of HD)^134^. Moreover, data from multiple HD rodent models as well as from post-mortem HD brain tissues suggest that the accumulation of mutant HTT in the nucleus is way more frequent in astrocytes and oligodendrocytes than in the microglia^135,136^. Corroborating the pathogenic effect of mutant HTT accumulation in cells other than the microglia, selective inactivation of mutant *HTT* in NG2^+^ oligodendrocyte progenitors prevents myelin abnormalities and certain behavioral deficits in HD mice^137^. Moreover, the cell-specific downregulation of mutant HTT from astrocytes or neurons coupled with modern sequencing technologies reveal that astrocyte dysfunction has a smaller impact on the neuronal transcriptome than neuron dysfunction has on the astrocytic one^138^. In line with this notion, at least three different transcriptional clusters of disease-associated astrocytes have been documented in post-mortem cingulate cortex samples from patients with HD, including a cluster with prominent stress-responsive and reactive transcriptional profile^139^. Transcriptional data from two distinct mouse models of HD and post-mortem HD brains also delineate the existence of shared transcriptional alterations linked to astrocytic dysfunction that can be corrected by limiting mutant HTT expression^140^. Taken together, these observations highlight the prominent role of inflammatory microglial activation as a driver of cellular dysfunction in the context of HD.

Of note, mutant HTT levels in circulating leukocytes have been shown not only to positively correlate with disease burden in patients with HD, but also to elicit immunological dysfunction coupled with increased TNF and IL8 secretion downstream of altered NF-κB functions^141^. In this setting, HTT silencing by RNA interference was sufficient to reverse secretory and transcriptional alterations^141^. Whether such an intervention would modify disease course in mouse models of HD, though, remains to be further investigated. That said, signs of both central and peripheral immune activation have been documented for both dendritic cells (DCs)^142^ and macrophages^143^ in R6/2 mice and zQ175 mice. Moreover, mutant *HTT* carriers exhibit a cerebrospinal T cell compartment characterized by increased IL17 expression coupled with the acquisition of a T_H_17 polarization and elevated IL7 consumption prior to symptom onset, and the abundance of cerebrospinal T_H_17 cells negatively correlates with disease progression^144^. These latter observations suggest that T cells may be involved in the early pathogenesis of HD and hence that limiting T cell responses may delay the onset of symptomatic HD.

**Fig. 3:**
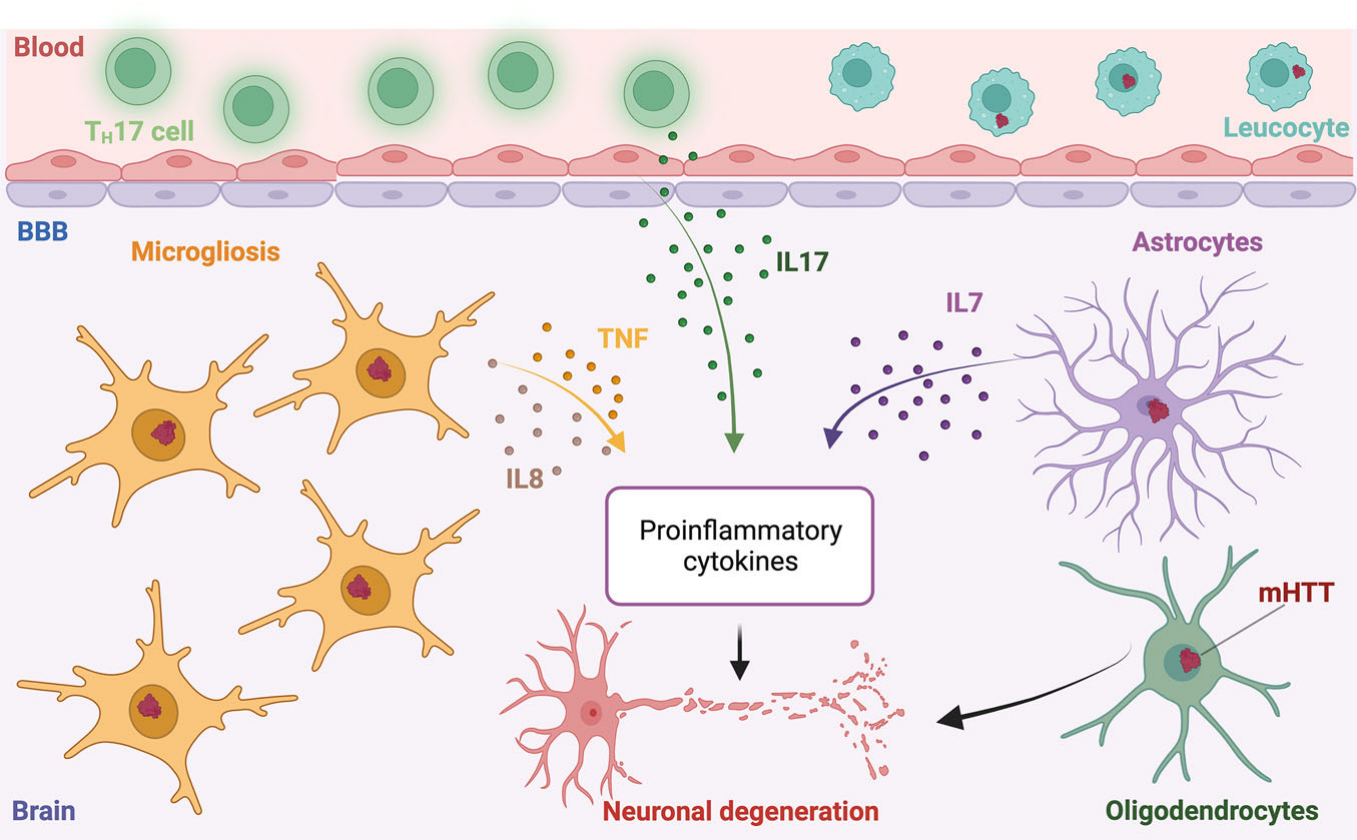
Immunological aspects of Huntington’s disease. Huntingtin (HTT) defects as caused by the mutations that drive Huntington’s disease (HD) foster a robust neuroinflammatory process involving microglial cells as well as astrocytes and oligodendrocytes that is often associated with a T_H_17-polarized CD4^+^ T cell response dominated by the secretion of pro-inflammatory cytokines like interleukin 8 (IL8), IL17A and tumor necrosis factor (TNF). BBB blood brain barrier, mHTT mutant huntingtin. Created with BioRender.com.

Thus, similar to AD and PD, HD appears to develop in the context of complex innate and adaptive immune responses that manifest both centrally and in the periphery (Fig. 3).

## Others

### Dementia with Lewy bodies

Dementia with Lewy bodies (DLB) is a prevalent form of dementia in the elderly that is defined by cognitive impairment coupled with visual hallucinations, parkinsonism, sleep behavior disorders, as well as autonomic and psychiatric dysfunction^145^. Of note, both AD and PD not only share familiar risk factors with DLB, notably genetic variants of *APOE* and *SNCA*^145^, but also exhibit similar clinical manifestations, making differential diagnosis problematic^146,147^. Moreover, at least a fraction of DLB cases share with AD the deposit of Aβ plaques as well as tau hyperphosphorylation, and with PD the accumulation of SNCA aggregates (Lewy bodies), although this does not often involve the SN, but instead affects basal ganglia and the cerebral cortex^145^. Finally, DLB has been associated with a history of traumatic brain injury (TBI), at least potentially linked to neuroinflammation^148,149^. However, the pathogenesis of DLB remains poorly understood, partly due to a lack of precise cellular and animal models of the disease.

Analysis of post-mortem brains from patients with DLB revealed diffuse cerebral inflammation and an increased number of microglial cells in the proximity of Lewy bodies^150^, as well as increased levels of proinflammatory cytokines such as IL6 coupled to the downregulation of neurotrophic factors^151^. Similar to the case of AD, microgliosis as associated with DLB appears to occur early during disease pathogenesis^152^ and to decline over time as cognitive impairment emerges^153^. Moreover, DLB has been associated with an increase in the circulating levels of several proinflammatory cytokines including IL2, IL17A and C-C motif chemokine ligand 20 (CCL20) along with decreased IL8 concentrations^153^, pointing to an involvement of both innate and adaptive immune mechanisms in the pathogenesis of the disease.

Further supporting a pathogenic role for immune effectors in the progression of DLB, CD4^+^ (but not CD8^+^) T cells have been shown to infiltrate the brain parenchyma of patients with DLB and SNCA-expressing mice, a rodent model of the disease that also manifests intracranial accumulation of natural killer T (NKT) cells^154^, a small lymphoid cell population with potent reactive traits^155,156^. Intriguingly, DLB-associated CD4^+^ T cell brain infiltration appears to be maximal in the proximity of blood vessels^154^, and these cells appear to acquire a T_H_17 phenotype coupled with pathogenic IL17A secretion in the cerebrospinal fluid downstream of C-X-C motif chemokine ligand 12 (CXCL12)-driven, C-X-C motif chemokine receptor 4 (CXCR4)-mediated recruitment^157^, a potent chemotactic signalling axis^158^.

These findings suggest that, while the precise etiology of DLB remains to be clarified, adaptive immune effectors including IL17A-secreting CD4^+^ T cells may contribute to DLB establishment and progression.

### Frontotemporal dementia

Frontotemporal dementia (FTD) is an early-onset neurodegenerative disorder driven by a progressive atrophy of the frontal and temporal lobes and characterized by alterations in behavior, impulse control, personality, and language^159^. Pathologically, FTD is characterized by abnormal accumulations of TAR DNA binding protein (TARDBP), hyperphosphorylated tau or FET proteins, which encompass EWS RNA binding protein 1 (EWSR1), TATA-box binding protein associated factor 15 (TAF15) and FUS RNA binding protein (FUS)^159^. Mutations in C9orf72-SMCR8 complex subunit (*C9Orf72*), *MAPT*, *TREM2* and granulin precursor (*GRN*) have been associated with familial variants of the disease^160–163^, but sporadic FTD accounts for > 60% of FTD cases^164^. Interestingly, FTD-related mutations have also been associated with an increased risk for autoimmune conditions^165,166^, pointing to a potential role for neuroinflammation in the pathogenesis of this ND.

In line with this possibility, FTD has been consistently associated with microgliosis and neuroinflammation in patients^167–169^. Moreover, individuals affected by FTD exhibit increased cerebrospinal levels of multiple pro-inflammatory cytokines including (but not limited to) IL2, IL12, IL17A, TNF, TGFB1 and CXCL1, at least in some patient cohorts positively correlated with disease severity^170–172^. Interestingly, cerebrospinal alterations in cytokine levels appear to vary in sporadic vs familial FTD cases^173^, but the causes underlying the observations remain to be determined. One study also identified a decrease in circulating B cells in patients with FTD^174^, but these findings await validation in larger patient cohorts.

Of note, *Grn*^*−/−*^ mice exhibit pro-inflammatory microglial activation downstream of NF-κB signaling^175,176^ coupled with the acquisition of an FTD-associated microglial state that: (1) is different from the AD- and ALS-associated DAMs, and (2) actively supports neurotoxic TARDBP granule deposition^177^, at least in part as a consequence of lysosomal dysfunction^178^. Along similar lines, mice expressing an FTD-associated TREM2 mutant exhibit brain-wide alterations including a delayed resolution of neuroinflammatory responses and a reduction of cerebral blood flow that may support disease progression^179^.

Vascular dysfunction and astrocytosis have also been observed in the frontal and temporal lobes of patients with FTD^180^. Specifically, a highly conserved astrocytic phenotype promoting synaptic degeneration and TARDBP neuropathy has been identified in the thalamus and frontal cortex of patients with GRN-associated FTD and *Grn*^*−/−*^ mice^181^, pointing to a central role for these cells in FTD progression.

In summary, FTD also involves an immunological component, although it remains poorly characterized. Additional studies are required to elucidate innate and potentially adaptive immune mechanisms involved in the pathogenesis of FTD.

## Conclusions

While most central NDs appear to originate from genetic or environmental alterations of cellular homeostasis in the brain parenchyma, it is now clear that such perturbations are accompanied by the activation of innate and (at least in some cases) adaptive immune effector mechanisms that contribute to disease pathogenesis. As abundantly discussed herein, multiple NDs are associated with mutations in genes encoding components of the innate or adaptive immune system, such as TREM2^29–31^ or HLA-DRB1^35^. Moreover, hitherto unrecognized connections are emerging between central ND susceptibility genes, such as *SNCA*, and core immunological functions, such as the development of normal innate and adaptive immune reactions to bacterial challenges^120^. Finally, patients affected by numerous NDs including AD, PD, HD, DLB and FTD exhibit shifts in the circulating levels of pro-inflammatory cytokines or peripheral immune populations, further supporting a pathogenic role for altered immune responses in the central nervous system in the progression of NDs. With a few exceptions including the robust implication of CD4^+^ in disease pathogenesis in mouse models of DLB^157^, most of the current links between immunological mechanisms and ND pathogenesis rely on observational and correlative rather than mechanistic experimental setups. While at least partially this reflects the limited number of rodent models that recapitulate the emergence and progression of NDs in humans, it will be important to harness currently available models to implement antibody-mediated depletion, pharmacological inhibition or genetic deletion/downregulation experiments to mechanistically link altered immune functions to ND pathogenesis and potentially identify novel targets for therapeutic interventions.

In the era of cancer immunotherapy (Box 2), the data summarized herein point indeed to the possibility of harnessing immunomodulatory agents beyond general anti-inflammatory and immunosuppressive drugs such as corticosteroids for the management of multiple NDs. While as mentioned above preclinical data in support of this possibility suffer from an overall observational nature, it is still tempting to postulate that currently approved therapeutics affecting immune functions may be beneficial for at least some patients with NDs. For instance, circulating IL17A elevations and/or polarization of the CD4^+^ T cell compartment toward a T_H_17 profile have been detected in patients with PD^144^, DLB^153^ and FTD^172^, and no less than three distinct IL17A blockers are currently available for the treatment of inflammatory conditions such as psoriasis^182^. Along similar lines, T_REG_ cells have been demonstrated to limit disease progression in multiple mouse models of AD^89–91^, pointing to adoptive T_REG_ transfer as an intriguing possibility to control AD progression in humans. Finally, PD-1 blockage has been associated with neuroprotective effects in mouse models of AD^81^, and multiple immune checkpoint inhibitors targeting PD-1 or its main ligand CD274 (PD-L1) are currently licensed for use in patients with various malignancies^24^.

Importantly, the use of immunomodulatory agents for the management of NDs has begun to be explored in the clinic. Specifically, a TREM2-targeting monoclonal antibody (AL002) is currently being assessed in patients with AD (NCT05744401)^39^, while an inflammasome inhibitor (RO7486967) is under investigation in individuals with PD (NCT05924243). Whether these or other immunomodulators are effective and will ultimately be approved for use in humans, however, remains to be established.

In summary, while additional work is required to elucidate the actual therapeutic potential of immunotherapy for patients with central NDs, both innate and immune dysfunctions have been documented during the progression of AD, PD, HD, DLB and FTD. It will be important to obtain further mechanistic insights into the immunological aspects of human degeneration in existing and newly developed rodent ND models to develop disease-modifying treatment options for these patient populations.

Box 2 Principles of anticancer immunotherapy While historically cancer has been viewed as a purely cell-intrinsic disease driven by genetic or epigenetic alterations that would confer malignant cell precursors with a survival or proliferative advantage over their normal counterparts, it is now clear that developing neoplasms must evade recognition by the host immune system to become clinically manifest^24,195^. Thus, most (if not all) clinically detectable tumors have acquired phenotypic features that allow them to go unrecognized by innate and adaptive immune cells or to resist their attack^196^. For instance, multiple tumors ultimately lose the expression of key proteins involved in antigen presentation, such as beta-2-microgoblulin (B2M), hence becoming virtually invisible to CD8^+^ cytotoxic T lymphocytes^197^ or express increased amounts of immunosuppressive molecules, such as CD274 (best known as PD-L1), thus actively suppressing T cell activation in the tumor microenvironment (TME)^198^. At odds with conventional anticancer treatments, which for the most part aim at directly killing cancer cells while sparing as much as possible healthy tissues, anticancer immunotherapy has been developed as an approach to restore the recognition and elimination of malignant cells by the host immune system^24^. While numerous forms of immunotherapy have been developed over the past century, including rather untargeted approaches (e.g., the systemic delivery of immunostimulatory cytokines such as IL2) as well as highly specific interventions (e.g., therapeutic vaccination based on one or several tumor-specific antigens)^199^, only a few of these approaches are approved by regulatory agencies and routinely employed in cancer patients^24^. Perhaps the most successful form of anticancer immunotherapy, which is commonly known as immune checkpoint blockade, relies on monoclonal antibodies that interrupt inhibitory signals provided by cancer cells or other cells of the TME to cytotoxic CD8^+^ T lymphocytes, including signals elicited by PD-L1^24^. Indeed, no less than 6 distinct immune checkpoint inhibitors (ICIs) are currently approved for use in more than 40 oncological indications^24^. It should be noted that multiple conventional anticancer treatments including some cytotoxic chemotherapeutics^200^, targeted anticancer agents^201,202^ as well as radiation therapy (at least when used focally and according to specific dose and fractionation schedules)^203,204^, have been shown to mediate therapeutically relevant immunostimulatory effects, which at least in part blurs the traditional discrimination between standard anticancer regimens and immunotherapy.
